# Sole vibration improves locomotion through the recovery of joint movements in a mouse cast model

**DOI:** 10.1371/journal.pone.0186189

**Published:** 2017-10-17

**Authors:** Atsushi Doi, Kazuaki Miyamoto, Yu-shin Nakano, Juntaro Sakasaki, Syota Kasae, Keisuke Nishimura, Min-Chul Shin, Megumu Yoshimura

**Affiliations:** 1 Department of Rehabilitation, Kumamoto Health Science University, Kumamoto, Kumamoto, Japan; 2 Graduate School of Health Science, Kumamoto Health Science University, Kumamoto, Kumamoto, Japan; 3 Department of Rehabilitation, Hitoyoshi Rehabilitation Hospital, Hitoyoshi, Kumamoto, Japan; 4 Department of Rehabilitation, Okabe Hospital, Umi-machi, Fukuoka, Japan; 5 Department of Rehabilitation, Tokyo-Wangan Rehabilitation Hospital, Narashino, Chiba, Japan; 6 Department of Rehabilitation, Shimizu Hospital, Kyoto, Kyoto, Japan; 7 Department of Rehabilitation, Iizuka Hospital, Iizuka, Fukuoka, Japan; 8 Department of Orthopedic Surgery, Nakamura Hospital, Nogata, Fukuoka, Japan; Tokai University, JAPAN

## Abstract

We investigated the effects of a vibratory stimulus on the plantar surface of the hind limb for motor, sensory, and locomotive function using a mouse cast model. The right knee joint of C57BL/6 male mice (7 weeks, 20 g, n = 31) was flexed with aluminum splint and tape for 6 weeks. These mice were randomly divided into 2 groups (control group, n = 11 and vibration group, n = 12). The mice in the vibration group received vibration on the sole of the ankle for 15 minutes per day, 5 days per week. After the knee joint cast was removed, we measured the range of motion (ROM) of both knee and ankle joints and the sensory threshold of the sole. Further, both walking and swimming movements were analyzed with a digital video. The sole vibration did not affect the passive ROM of the knee joint and sensory threshold after cast removal. However, it increased the ankle dorsiflexion range and improved free walking, swimming, and active movement of the knee joint. In conclusion, we show that the vibration recovered both walking and swimming movements, which resulted from improvements in both the passive ankle dorsiflexion and active knee movement.

## Introduction

Casting has been utilized as one of the standard methods for stabilizing the joints and soft tissues in post-traumatic injuries [1–3]. However, after cast removal, several joint problems develop, such as limitation of range of motion (ROM) [4], muscle weakness [5], and mechanical hyperalgesia [6]. Therefore, medical rehabilitation is needed to resolve these problems [7]. In addition to rehabilitative training, recent physical therapy methods such as electrical and vibratory stimuli [8,9] have been used to facilitate recovery from joint problems.

Regarding basic research of the vibratory stimulus for immobilization rat model, papers have reported that local vibration affect sensory function [10]. Further, in terms of clinical research of the vibratory stimulus for patients, such as chronic obstructive pulmonary disease, and stroke, a couple of papers have already stated that both whole-body and local vibration affects motor performance [11,12]. However, the effects of local vibration on joint movement, sensory threshold, and behavior for immobilized animal condition need further clarification.

In this study, we aimed to investigate the effects of vibratory stimuli on the plantar surface of the hind limb of a mouse cast model in terms of motor, sensory, and locomotive function.

## Materials and methods

### Animals

Male C57BL/6J mice (5 weeks old, 20–23 g, n = 31) were purchased from Kyudo, Inc. (Kumamoto, Japan) and housed under controlled temperature (24 ± 1°C) and humidity (55 ± 10%), with a 12-hour light-dark cycle and with food and water freely available. The animal experiments were approved by the Animal Care Committees of Kumamoto Health Science University (approval No. 14–015) and were conducted in accordance with the National Institute of Health guide for the care and use of laboratory animals (NIH Publications No. 80–23, revised 1996).

Briefly, the mice were anesthetized with sodium pentobarbital (50 mg/kg). Under anesthesia, the right knee joint was maximally flexed and wrapped with aluminum fence splint (Alcare, Tokyo, Japan) and tape (Protector Refle, Daiwa-Kan, Japan). After the knee joint was immobilized, we carefully checked whether the adjacent ankle joint had normal motion. If any problems were noted with the ankle, such as joint limitation or foot edema, we released the wrapping and re-wrapped the joint. In this study, we performed three sets of experiments for which cast mice were divided into two groups (control group and vibration group). The first set of experiments involved 2 weeks of casting and only knee extension was evaluated (control group, n = 4; vibration group, n = 4). The second set of experiments lasted 6 weeks and we measured knee extension, ankle range of motion (ROM), and sensory disturbance and analyzed the free walking of mice (control group, n = **6;** vibration group, n = **6**). The third set of the experiments involved 6 weeks of casting, and we measured ankle ROM and analyzed walking, footprints during walking, and swimming in mice (control group, n = 5; vibration group, n = 6).

### Vibration

A vibratory stimulus was provided to the mice while their right knee joint was wrapped in a splint with tape. The vibration (frequency, 4900 times per minute) was performed using HB-M01-A (Ohm Electric Inc., Tokyo, Japan), which is commercially available at low cost. At first, the casted mice were fixed with a plastic tube while awake. The cast was then further clamped with an adjusting magnetic base and stand (A-2, Shinwa Rules Co, Sanjyo, Niigata, Japan) and lab clamp (NC-3, Kenis, Osaka, Japan). The vibration equipment was held with another adjusting magnetic base and stand (A-2, Shinwa Rules Co, Sanjyo, Niigata, Japan), and lab clamp (NC-3, Kenis, Osaka, Japan), whose tip was attached to the distal and plantar surface of the foot. Stimuli in the vibration group were delivered for 15 minutes, 5 times per week, for 2 weeks (first set of experiments) or 6 weeks (both second and third sets of experiments). In the control group, vibration equipment was attached to the foot for 15 minutes, but without actual vibratory stimulus.

### Measurement of both knee and ankle joints

The ROM of the knee joints was measured twice per week after cast removal. For the measurement of joint movement, a plastic tube was fixed to the mouse and the mouse was laid on its side in a plastic box in the sodium pentobarbital anesthetized condition (50 mg / kg). The reason we used a plastic tube under the anesthetized condition was to fix the trunk for the measurement of the knee ROM. Thereafter, an iPhone 5S (Apple, Inc.) was placed on top of the plastic box. After photographs of the maximum ROM of knee extension, knee flexion, ankle dorsiflexion, or ankle extension had been taken, these ROMs were measured with ImageJ software. In particular, when both knee flexion and extension were measured, bamboo skewers were placed on the thigh and lower leg as markers.

### Sensory disturbance

Sensory threshold was measured with an electrical stimulation of 2000 Hz (STG—4002, Multichannel Systems Inc, Reutlingen, DE, Germany). A plastic tube was affixed to the mouse, ball-type bipolar electrodes were placed on the plantar surface of the foot, and electrical stimulation was applied. The electrical stimulation-induced withdrawal reflex of the mouse hind limb resulted in an avoidance of the electrode. After the time from the beginning of the electrical stimulation to the appearance of the withdrawal reflex was measured, we calculated the intensity (μA) of the stimulation.

### Behavior analysis

**Fig 1:**
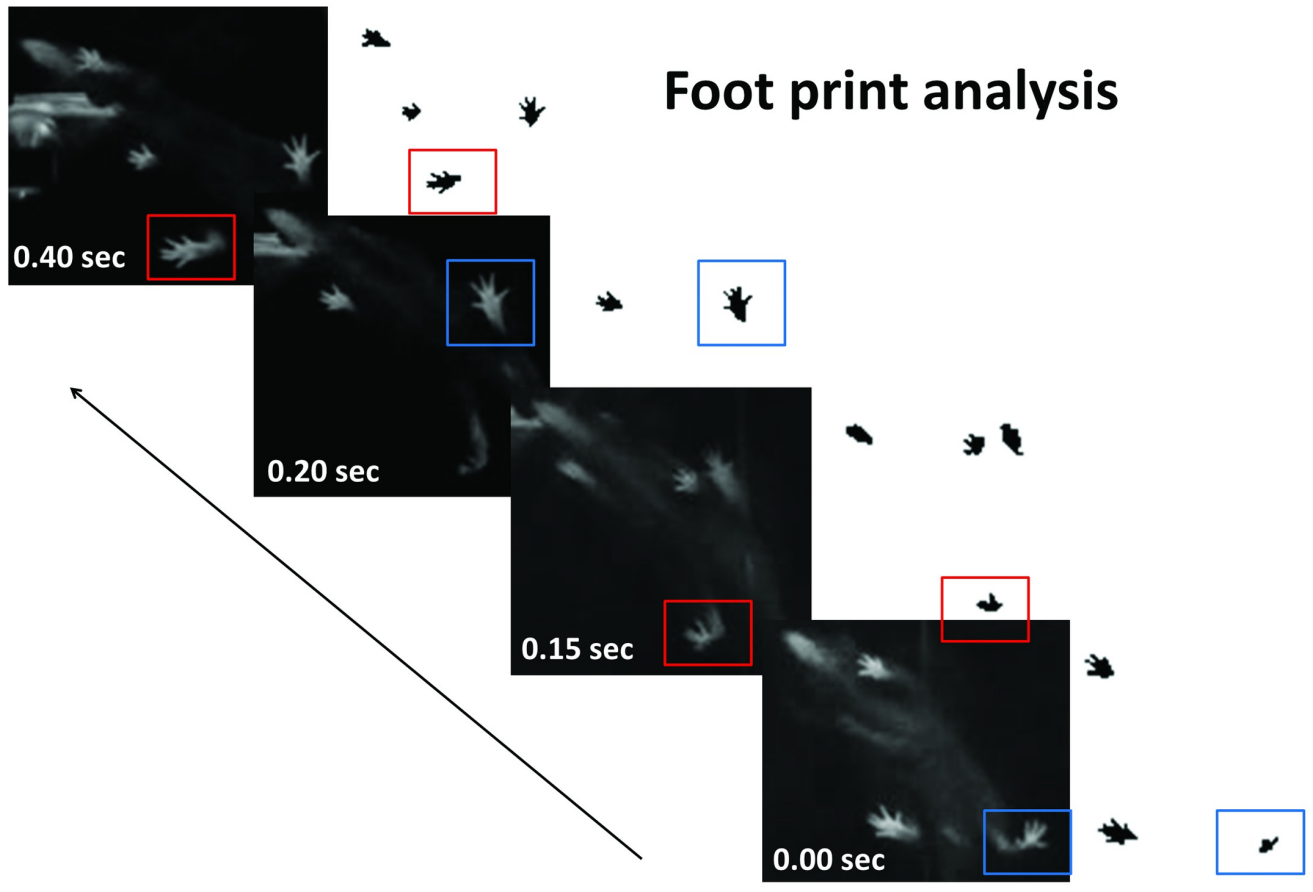
Footprint analysis. Footprints of the right (red square) and left (blue square) hind limbs were analyzed.

All the video recordings for both walking and swimming were performed 7 weeks after the joint cast was removed. Regarding mouse free walking, the movement during free walking was recorded with a digital camera (20 frames/sec, TZ—35, Panasonic, Japan) from above and below in a 40 cm x 40 cm plastic box. While tracking walking from above the mouse, tracking shape (straight, small circle, long circle, and square), direction of walking (only clockwise, or clockwise and anticlockwise), walking distance (cm/40 s), maximum speed (cm/s), average speed (cm/s) were measured (n = 11 and 12). While tracking walking from below the box in which the mouse was kept, swing phase, stance phase, paw contact area in the stance phase, and stride length were measured (n = 5). To detect the mice’s paw from below, the plastic box was first covered with black sheets and we placed a faint light below the box. Each mouse walked 10 steps in the 40 cm x 40 cm plastic box. The examiner confirmed the swing and stance phase on the PC screen. The time was measured by checking each frame (0.05 frame/sec). Not only the average of the swing and the stance phase at 10 steps but also the ratio of the swing phase to the stance phase was calculated. The paw contact area was measured with Image J and was analyzed as the ratio of the cast side (right) to the non-cast side (left) area. Stride length was analyzed as the ratio of the right to the left stride length (Fig 1).

**Fig 2:**
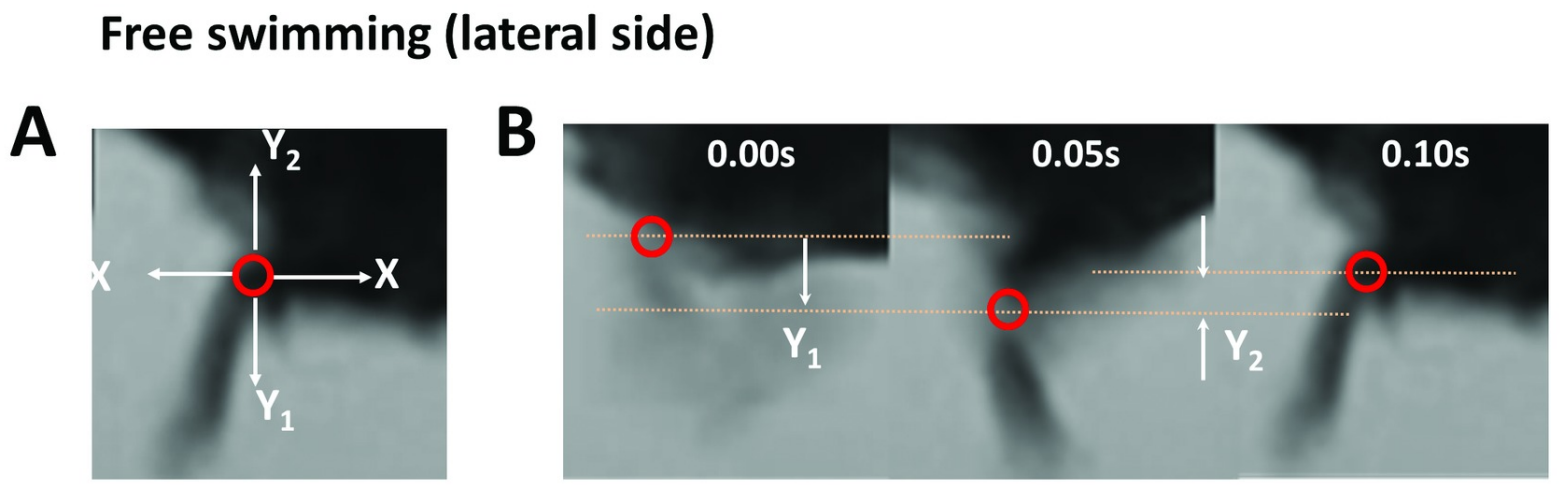
Analysis of the swimming at the lateral side. Photographs of the swimming analysis of the lateral side (Aa and Ab).

Free swimming was also recorded with the digital video camera from both the upper and lateral side. For the upper side, the direction of turn movement, straight swimming, swimming distance (cm / 40 s), maximum speed, (cm / s), and average speed (cm / s) were measured. For the lateral side, we only focused on the longitudinal (Y) axis because the mouse moves in the direction of the side (X) axis on the lateral side (Fig 2).

After the distal end of the right tibia (ankle joint) was identified using the frame-by-frame of each recorded video data frame, the tibial end was marked on each frame. The distances Y, Y1, and Y2 were measured (Fig 2). To analyze the videos of walking and swimming, we used Avidemux (http://fixounet.free.fr/avidemux/), Any Video Converter (http://www.any-video-converter.com/products/for_video_free/), VirtualDub (http://www.virtualdub.org/), and ImageJ (https://fiji.sc/), which are open source software. During the first step of the analysis, these videos were cut and edited with Avidemux, Any Video Converter, and VirtualDub. Thereafter, behaviors were analyzed with ImageJ.

### Statistical analysis

Experimental data are expressed as mean ± SEM. Single comparisons were conducted using Wilcoxon’s signed-rank test for paired groups and the Mann-Whitney U-test for unpaired groups or chi-square test for data distribution of unpaired groups. p < 0.05 was considered statistically significant. All statistical analyses were performed with EZR (Saitama Medical Center, Jichi Medical University, Saitama, Japan), which is a graphical user interface for R (The R Foundation for Statistical Computing, Vienna, Austria). More precisely, it is a modified version of R commander designed to add statistical functions frequently used in biostatistics [13].

## Results

### The sole vibration did not affect the passive knee angle after cast removal

**Fig 3:**
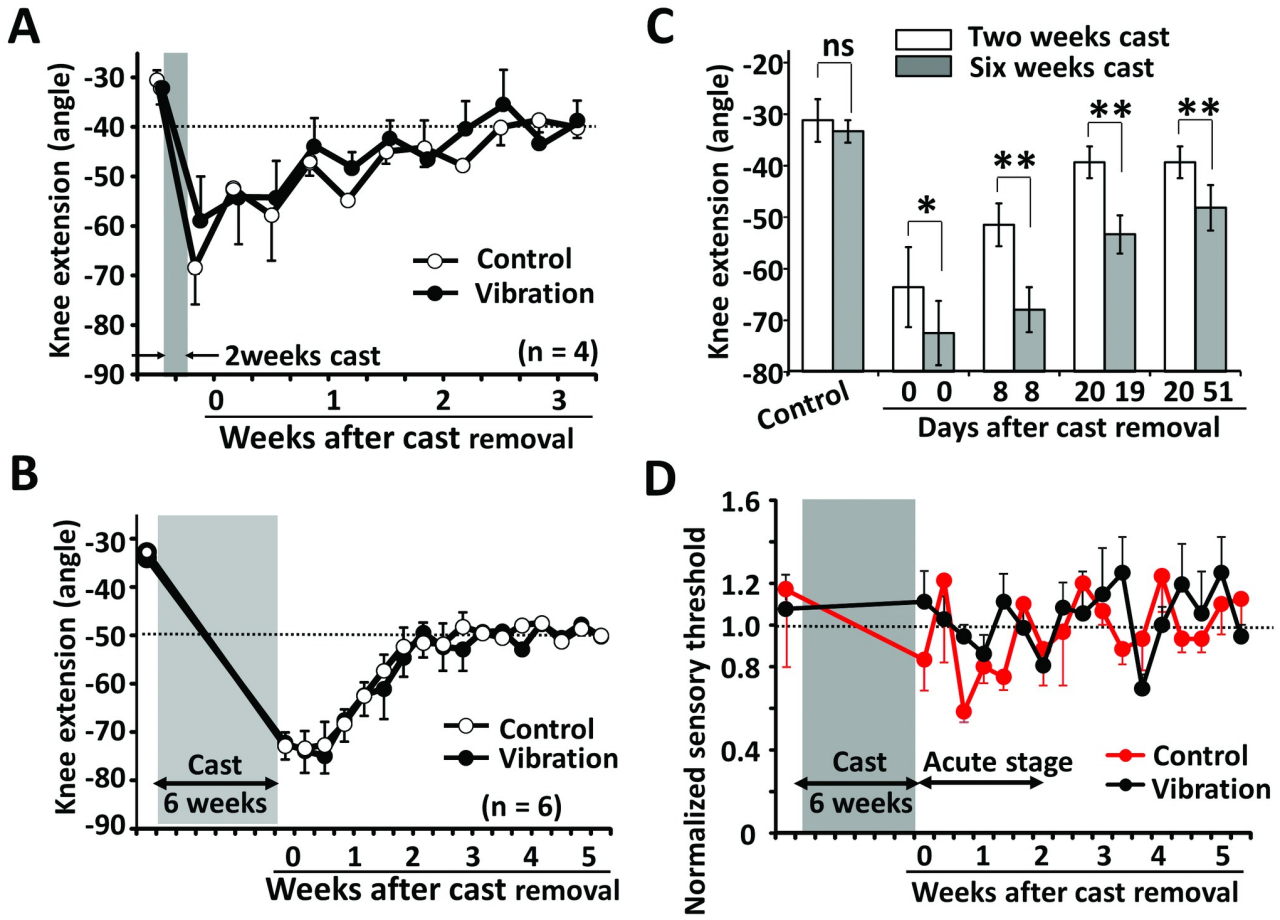
Knee extension angle after removal of casting, and the changing of the sensory threshold. (A) Knee extension angle at 2 weeks after casting. (B) Knee extension angle at 6 weeks after casting. (C) Comparison between 2-week and 6-week casting. White and black circles show the control and vibration groups, respectively. (D) The changing of the sensory threshold between the control and vibration groups.

In both the control and vibration groups, the angle of knee extension reached approximately -40° within 3 weeks of the removal of the 2-week casting (Fig 3A). However, there was no statistical difference in knee extension between both groups (Fig 3A), suggesting that the duration of casting might be a major factor for the indication of ankle vibration therapy. Therefore, the duration of the casting was changed from 2 weeks to 6 weeks. Although the knee angle slowly recovered after the removal of the 6-week casting, the recovery process of the knee angle between these 2 groups was fundamentally the same (Fig 3B). Notably, the knee extension angle after the 2-week casting was significantly smaller than that after the 6-week casting (Fig 3C). These results show that the sole vibration itself did not affect the knee angle after the removal of the knee cast.

### The sole vibration may not have contributed to touch sensation after cast removal

We investigated whether the vibration contributed to the recovery of the sensory threshold. At the acute stage (within 2 weeks). After the 6-week casting, the 2000-Hz electrical stimulation-induced sensory threshold in the control group tended to decrease (Fig 3D, red line), suggesting that temporal hypersensitivity in the control group may be caused on the plantar side of the cast limb. The electrical stimulation-induced sensory threshold in the vibration group did not decrease notably (Fig 3D, black line), However, there was no statistical difference in sensory threshold between these 2 groups (ns: p = 0.924, Fig 3D).

### The vibration improved free walking

**Fig 4:**
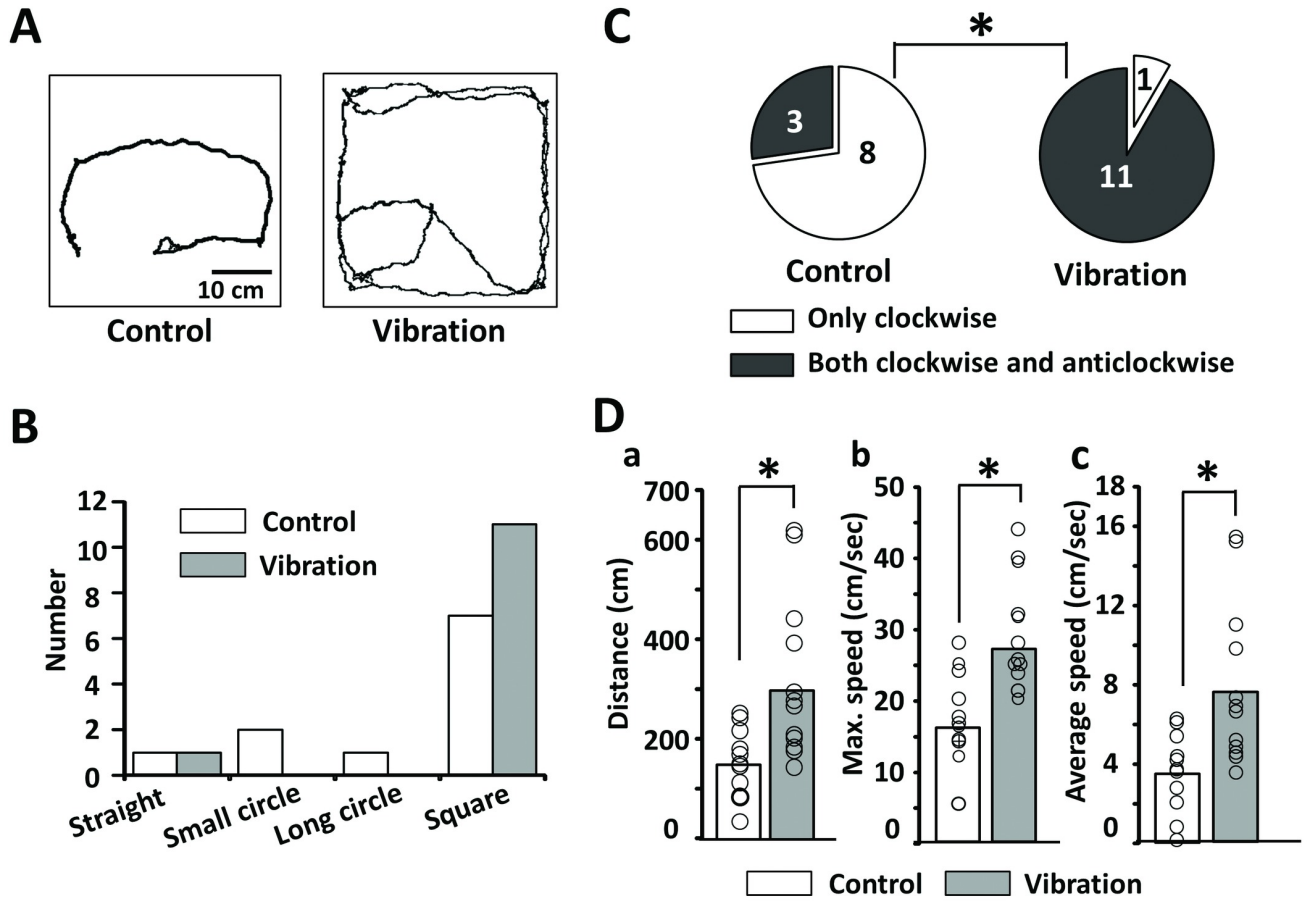
Gait analysis of the upper side. **(A)** Gait tracking between the control and vibration groups. (B) Classification of gait tracking shapes in the control and vibration groups. (C) Direction of the turn movement in the control and vibration groups. Comparison of gait distance (Da), maximum speed (Db) and average speed (Dc) between control and vibration groups (* p = 0.002–0.003).

Next, we analyzed several parameters, such as tracking shape, direction of the turn movement, gait distance, maximum speed, and average speed using the video data of the free walking. First, the tracking shape was compared between the control and vibration groups (Fig 4A). However, there were not many differences in the tracking shape between both groups (Fig 4B). Eleven in twelve cases in the vibration group could turn both clockwise and anticlockwise (Fig 4C). On the other side, 8 in 11 cases in the control group could only turn clockwise (* p = 0.002, Fig 4C). The gait distance (* p = 0.003, Fig 4Da), maximum speed (* p = 0.002, Fig 4Db), and average speed (* p = 0.003, Fig 4Dc) of the vibration group were significantly larger than those of the control group.

### The vibration changed the foot ground area in the stance phase of walking

**Fig 5:**
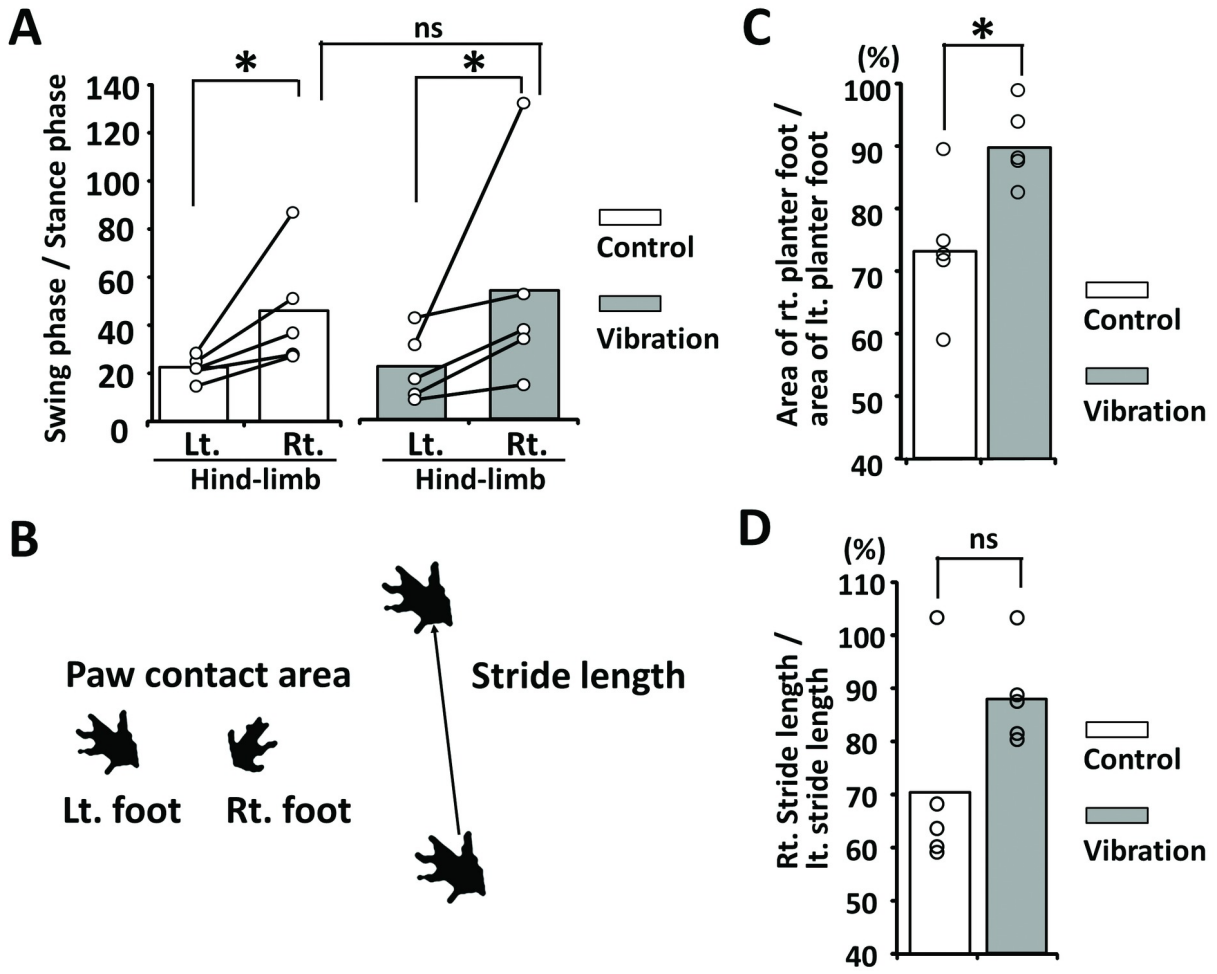
Footprint analysis in the control and vibration groups. (A) Ratio of the swing phase to the stance phase in the control and vibration groups. (B) The paw contact area and stride length. (C) Ratio of the right to the left plantar surface of the foot in the control and vibration groups. (D) Ratio of the right to the left stride length in the control and vibration groups.

Next, we analyzed the footprints of 5 cases (Fig 1). The duration of the swing phase was much longer on the right side than on the left side in both groups (* p = 0.03 for both control and vibration groups, Fig 5A). However, there was no statistical difference in the duration of the swing phase between both groups (ns: p = 0.754, Fig 5A). The ground area of the plantar surface of the foot on the right side was significantly smaller than that on the left side (* p = 0.03, Fig 5B and 5C). Stride length in 80% (4 in 5 cases) of the vibration group was significantly longer than that of the control group. However, the difference was not significant (ns p = 0.117, Fig 5B and 5D).

### The vibration increased the range of ankle dorsiflexion

**Fig 6:**
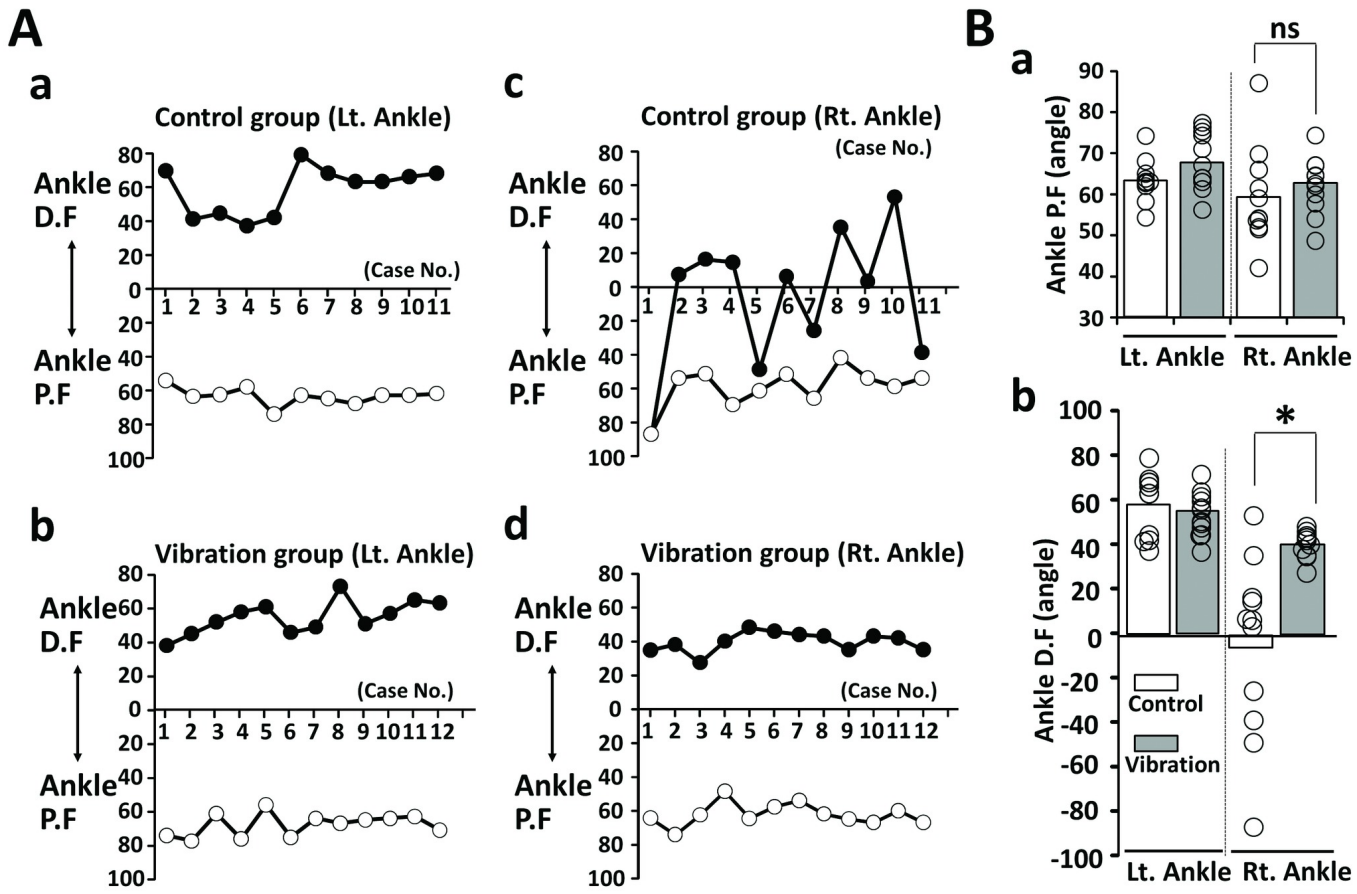
Range of ankle dorsal and plantar flexion in the control and vibration groups. (A) Range of ankle dorsal and plantar flexion in the control (n = 11) (Aa and Ac) and vibration groups (n = 12) (Ab and Ad). (B) Average ankle plantar flexion (Ba) and dorsal flexion (Bb).

To further investigate the improvement in free walking in the vibration group, we measured the passive motion of the ankle joint for both groups. The left ankle joint motion of both the control and vibration groups exhibited no differences (Fig 6Ba and 6Bb). Furthermore, planterflexion range of the right ankle in the vibration group was not significantly different from that of the control group (ns: p = 0.23, Fig 6Ba). However, the right ankle dorsiflexion range in the vibration group experienced greater increase compared to that of the control group (* p = 0.001, Fig 6Ac and 6Bb).

### The vibration improved free swimming

**Fig 7:**
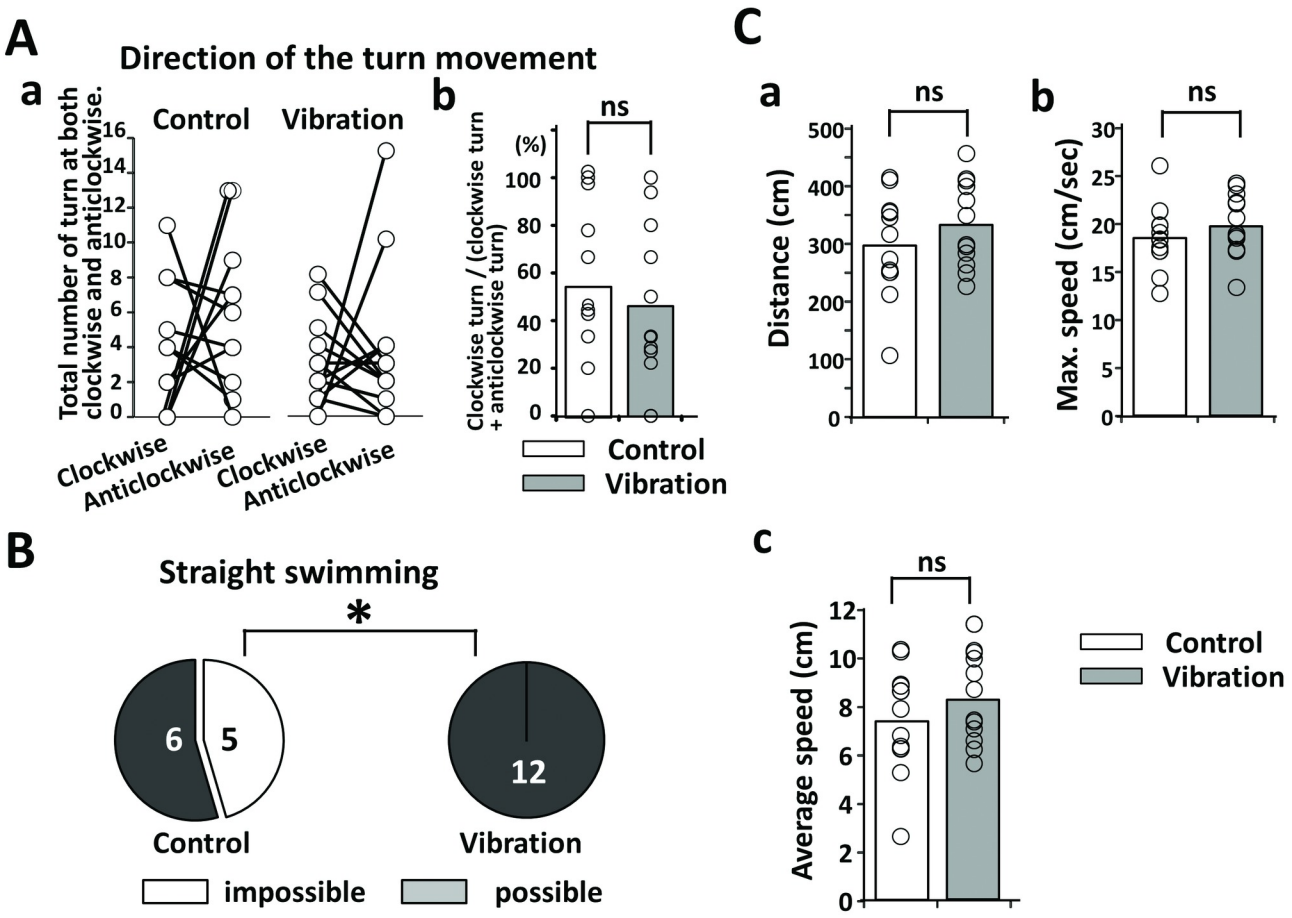
Swimming analysis of the upper side. (A) Direction of the turn movement in the control and vibration groups (control group: n = 11 and vibration group: n = 12) (Aa). Ratio of the clockwise turn to the total (clockwise plus anticlockwise) turn (Ab). (B) The frequency of the straight swimming. (C) Gait distance (Ca), maximum speed (Cb), and average speed (Cc) between control and vibration groups.

We analyzed the video data for the free swimming. First, the total numbers of turns in both clockwise and anticlockwise directions were compared between the control and vibration groups; however, the clockwise rate was not statistically different between groups (ns: p = 0.339, Fig 7Ab). Notably, all twelve cases in the vibration group achieved straight swimming (Fig 7B). However, 5 in 11 cases in the control group could not swim straight (* p = 0.01, Fig 7B). The gait distance, maximum speed, and average speed of the control and vibration groups did not differ statistically (ns: p = 0.371, Fig 7Ca, ns: p = 0.236, Fig 7Cb, and ns: p = 0.371, Fig 7Cc).

### The vibration may have improved active movement of the knee joint

**Fig 8:**
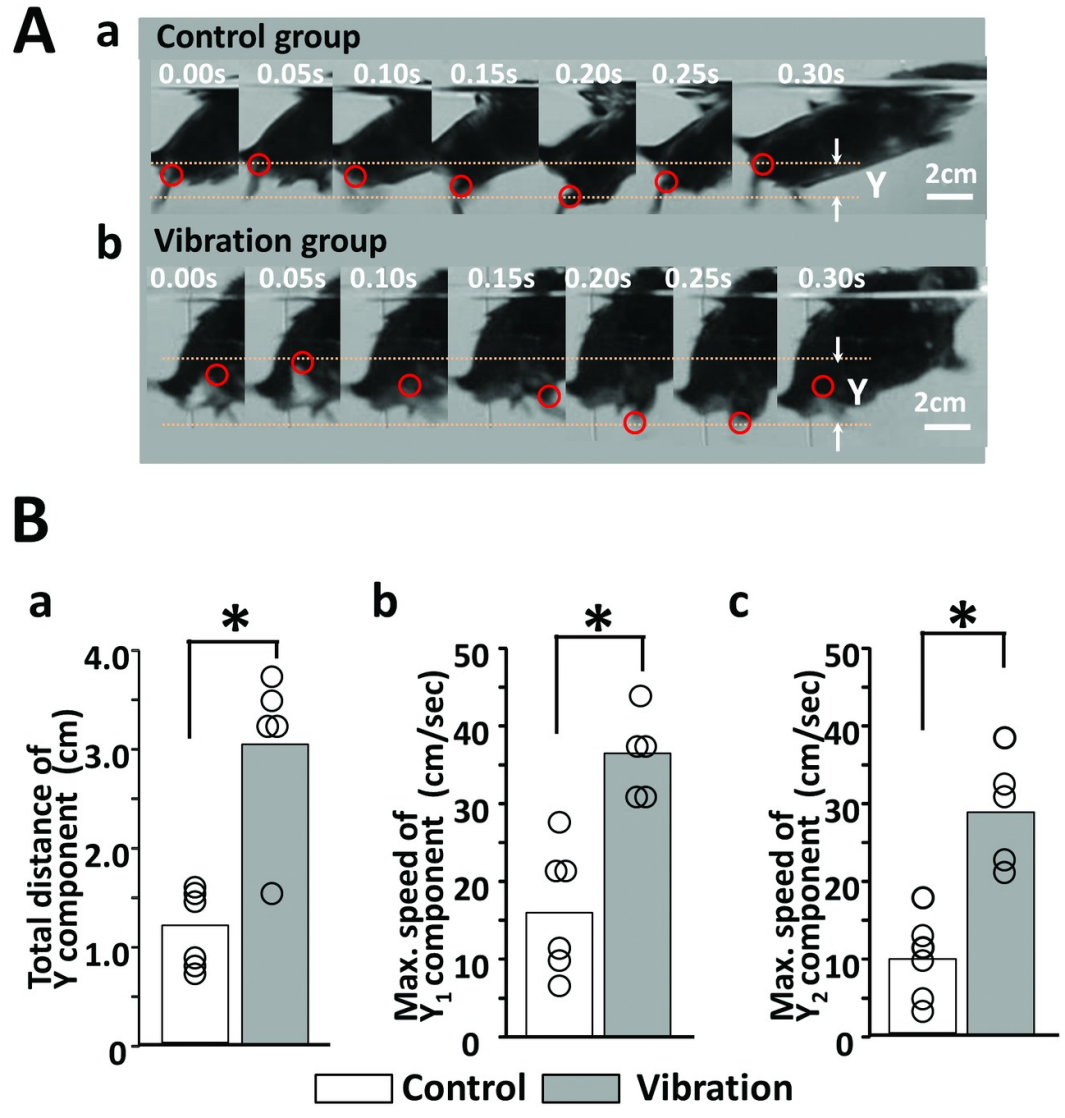
Swimming analysis on the lateral side. (A) Photographs of the control (Aa) and vibration groups (Ab). (B) Total distance of the Y component (Ba), maximum speed of the Y1 component (Bb), and maximum speed of the Y2 component (Bc).

As previously demonstrated, there was no difference in passive knee extension between the control and vibration groups after cast removal (Fig 3A and 3B). However, from the behavior of the mice, it was unclear how the knee joint was actively utilized. Further, during walking, the ankle joint always plays an important role in both reciprocal movement and in the stance phase of the walking. Therefore, we considered that utilizing walking alone may not be sufficient for analyzing the motion of the lower extremities, except for the ankle joint. Here, swimming activity on the lateral side was selected for the motion analysis of the active knee joint because the influence of the ankle joint could be removed. The total distance of the Y (Y1 plus Y2) component was significantly longer in the vibration group (n = 5) than in the control group (n = 6) (* p = 0.01, Fig 8A). Further, maximum speeds of both Y1 and Y2 components were statistically much higher in the vibration group than in the control group (* p = 0.007 at Y1 component, Fig 8B, * p = 0.004 at Y2 component, Fig 8C).

## Discussion

Regarding passive joint movements, sole vibration affected the ankle joint but not the knee joint. The reason for the limitation in ankle dorsiflexion after 2 and 6 weeks of casting is that just after the wrapping of the knee joint, the position of the ankle joint tends to be in plantar flexion due to gravity. This may explain the limitation in ankle dorsiflexion before the sole vibration was applied. Compared to the control group, which only had the vibration equipment attached to the sole for 15 minutes without the application of vibration, the vibration group showed statistically improved passive ankle movement (Fig 6Bb). Therefore, the sole vibration may have stretched the soft tissue, capsule, and ligaments of the ankle joint, and gastrocnemius muscle [14–16].

Previous studies have showed that immobilization in rodents causes hypersensitivity [17–20] and that continuous vibration or treadmill training partially recovers the hypersensitivity [21–23]. Further, Hamaue et al. claimed that only a 4-week immobilization resulted in hypersensitivity [18]. In this study, however, we did not observe any changes in the sensory threshold even without vibration (Fig 3D) [21]. The casting and non-weight-bearing conditions did not cause hypersensitivity in the acute stage after cast removal, even after 6 weeks of immobilization, probably due to the measurement of the sensory threshold. Moreover, Hamaue et al. used the von-Frey filament for threshold measurement [21], whereas, we utilized the electro-stimulus. Further, in this study, we only used 2000 Hz as the frequency for the electro-stimulation. According to previous papers, the 2000-Hz electro-stimulus mainly activates the Aβ fibers, which are related to the sense of touch [24]. If we had used other frequencies for electro-stimulation, hypersensitivity might have been demonstrated in the immobilized condition, and the vibration might have been affected, resulting in sensitivity change [17–21].

Although the sole vibration did not demonstrate any effects on passive knee extension (Fig 3), the vibration facilitated an active range of hind limb joint and maximum speed of the joint movement during swimming (Fig 8). However, we cannot assert that the hind limb motion resulted from the knee movement because the motion of the knee joint might not be detected accurately. However, since the marker of the ankle joint was placed on the lateral malleolus, we strongly suggest that sole vibration affected the active movement of the knee joint. Regarding the knee joint, the functional difference between active and passive movements may be due to a difference in muscle activation in both the quadriceps and hamstrings during knee movement [25]. Potentially, passive knee movement is not accompanied by muscle activity. However, during active knee movement, the contraction of either the quadriceps or hamstrings will need reciprocity [25]. Researchers have previously reported that whole-body vibration affects the activation of the quadriceps and the flexibility of the hamstrings [26–29]. They have also shown that Achilles tendon vibration could prevent atrophy of the soleus muscle in unweighted rats. Further, another report has shown that vibratory stimulus affects neuromuscular control via neuromuscular synapse protein changes following bed rest [30]. Thus, local vibration may act not only on muscle contraction and elasticity but also on neuromuscular junction-related proteins. Therefore, it may be necessary to evaluate the proteins and morphology of the thigh muscle of mice.

In conclusion, we showed that the vibration recovered both walking and swimming movement behaviors, which resulted from improvements in both the passive ankle dorsiflexion and active knee movement. Previous clinical studies show that vibration affects muscle strength [15,29] and balance [31–34], suggesting that in cases of human immobilization, sole vibration may prevent muscle atrophy and joint and/or muscle contracture.
